# Cathepsin B inhibition interferes with metastatic potential of human melanoma: an *in vitro *and *in vivo *study

**DOI:** 10.1186/1476-4598-9-207

**Published:** 2010-08-04

**Authors:** Paola Matarrese, Barbara Ascione, Laura Ciarlo, Rosa Vona, Carlo Leonetti, Marco Scarsella, Anna M Mileo, Caterina Catricalà, Marco G Paggi, Walter Malorni

**Affiliations:** 1 Department of Therapeutic Research and Medicines Evaluation, Istituto Superiore di Sanità, Viale Regina Elena 299, 00161, Rome, Italy; 2 Department of Experimental Chemotherapy, Regina Elena Cancer Institute, Via Elio Chianesi 53, 00144 Rome, Italy; 3 Development of Therapeutic Programs, Regina Elena Cancer Institute, Via Elio Chianesi 53, 00144 Rome, Italy; 4 Department of Dermatology-Oncology, S. Gallicano Dermatological Institute, Via Elio Chianesi 53, 00144, Rome, Italy

## Abstract

**Background:**

Cathepsins represent a group of proteases involved in determining the metastatic potential of cancer cells. Among these are cysteinyl- (e.g. cathepsin B and cathepsin L) and aspartyl-proteases (e.g. cathepsin D), normally present inside the lysosomes as inactive proenzymes. Once released in the extracellular space, cathepsins contribute to metastatic potential by facilitating cell migration and invasiveness.

**Results:**

In the present work we first evaluated, by *in vitro *procedures, the role of cathepsins B, L and D, in the remodeling, spreading and invasiveness of eight different cell lines: four primary and four metastatic melanoma cell lines. Among these, we considered two cell lines derived from a primary cutaneous melanoma and from a supraclavicular lymph node metastasis of the same patient. To this purpose, the effects of specific chemical inhibitors of these proteases, i.e. CA-074 and CA-074Me for cathepsin B, Cathepsin inhibitor II for cathepsin L, and Pepstatin A for cathepsin D, were evaluated. In addition, we also analyzed the effects of the biological inhibitors of these cathepsins, i.e. specific antibodies, on cell invasiveness. We found that i) cathepsin B, but not cathepsins L and D, was highly expressed at the surface of metastatic but not of primary melanoma cell lines and that ii) CA-074, or specific antibodies to cathepsin B, hindered metastatic cell spreading and dissemination, whereas neither chemical nor biological inhibitors of cathepsins D and L had significant effects. Accordingly, *in vivo *studies, i.e. in murine xenografts, demonstrated that CA-074 significantly reduced human melanoma growth and the number of artificial lung metastases.

**Conclusions:**

These results suggest a reappraisal of the use of cathepsin B inhibitors (either chemical or biological) as innovative strategy in the management of metastatic melanoma disease.

## Background

Cathepsins are a large family of cysteinyl-, aspartyl- and serine-proteases composed of at least twelve different molecules, which are distinguished by their structure, catalytic mechanism, and substrate specificity [1,2]. They are normally found inside the cell and appear commonly sequestered in well-defined organelles, mainly lysosomes, as inactive proenzymes [3]. When cathepsins are released outside the cell and activated, they trigger the degradation of the constituents of the extracellular matrix and basement membrane, such as type IV collagen, fibronectin, and laminin [4]. Their proteolytic activity has been suggested as a key factor in determining the metastatic potential of cancer cells [5]. Indeed, either cysteinyl- or aspartyl-proteases, by degrading the extracellular matrix, can directly contribute to cell migration and invasiveness, at least by dissolving the physical barriers limiting cell movements and spreading [for a review see [6]]. Among the members of this family of proteases, cathepsins B, D, K and L are hypothesized to play a major role [7,8].

Cutaneous melanoma arises from melanocytes and represents the most aggressive form of skin cancer. As for other cancers, melanoma progression is believed to depend upon a series of increasing survival-oriented molecular alterations correlated with the capability to generate a more malignant phenotype. The ultimate result of this process is the development of cancer cell clones selected for their ability to survive in extremely unfavorable microenvironmental conditions and capable of overwhelm the lack of nutrients and the deficiency of metabolic products. Indeed, despite chemo- and radio-therapeutic treatments, these cells can deceive host's immune response, survive hypoxia, oxidative stress, induction of apoptosis, and ultimately develop a remarkable propensity for metastatic spreading, the most life-threatening event in melanoma patients [9]. The key role of cathepsins in metastatic melanoma progression has been investigated in several experimental and clinical studies, where overexpression of cathepsins was associated with a worse prognosis and high cancer dissemination [10-13].

In the present work we investigated in both *in vitro *and *in vivo *systems the effects of cathepsin B, D and L inhibitors, i.e. chemical and biological (e.g. antibodies) in modulating metastatic melanoma cells invasiveness. We found that, unlike cathepsins D and L, the inhibition of cathepsin B significantly impaired cell invasiveness and metastatic potential.

## Materials and methods

### Melanoma cell cultures and in vitro treatments

The HLA-A2 1B6 (indicated as PM1 throughout the paper) and 8863 (indicated as MM1 throughout the paper) melanoma cell lines were obtained as previously reported [14-16]. The LP (PM2) cell line derived from a primary cutaneous melanoma (Clark's level V; Breslow 12 mm), while the LM cell line (MM2) from a supraclavicular lymph node metastasis of the same patient [17]. Other human melanoma cells used (PM3 and PM4 and MM3) were obtained from melanomas (primary or metastatic, respectively) of patients surgically resected at the Istituto Nazionale dei Tumori, Milan, Italy. Human M20 melanoma cell line (MM4) was obtained as reported [18]. All cell lines were cultivated at 37°C in 5% CO_2 _atmosphere in RPMI-1640 supplemented with 50 U/ml penicillin, 50 mg/ml streptomycin (BioWhittaker, Verviers, Belgium) and 10% FCS (Sebam, Berlin, Germany). For cell migration and invasion assays, cells were seeded in the presence of the cathepsin D inhibitor (Pepstatin A, 100 μM, Calbiochem), the cathepsin L inhibitor (cathepsin L inhibitor II, Z-FY-CHO, 10 μM, Calbiochem) as well as two cathepsin B inhibitors: CA-074 (cell unpermeant) and CA-074Me (cell permeant) (both given at a concentration of 10 μM, Calbiochem, Nottingham, UK). Since these two inhibitors gave similar results, i.e. not-significant differences were detected in all the experimental conditions considered here, only the results obtained with the CA-074 will be reported. Cells treated with the same concentration of the solvent (DMSO) were considered as control. Alternatively to these synthetic inhibitors, cells were also incubated with specific antibodies to cathepsin B, D (both Calbiochem) or L (Alexis) (60 μg/ml). Cells treated with the same concentration of IgG were considered as control.

### Relative quantification of procatepsin B gene expression by real-time RT-PCR

One-step quantitative RT-PCR analysis for procathepsin B and cyclophilin A expression was performed as described [19]. Quantitative RT-PCR for cyclophilin A was carried out on each sample as an internal control for template levels.

Primer sequences for procathepsin B:

FW: 5'- GATCATGTGGCAGCTCTGGGCCTCCCTCTG -3';

RV: 5'- GTCTTAGATCTTTTCCCAGTACTGATCGG -3'.

Primer sequences for cyclophilin A:

FW: 5'-TGGTCAACCCCACCGTGTTC-3';

RV: 5'-GCCATCCAACCACTCAGTC-3'.

Primer sequences were obtained from the NCBI database (GenBank accession number E10341.1 and BC000689, respectively).The relative expression of human procathepsin B mRNA was calculated as described [20].

### Analysis of lysosomal compartment

For evaluation of lysosomal acidity and volume, cells were stained with 1 μM LysoSensor probe or LysoTracker probe, respectively for 5 min (LysoSensor) or 15 min (LysoTracker) at 37°C and immediately analyzed by a cytometer. Comparisons among different melanoma cell lines (either primary or metastatic) were conducted by CellQuest Software using the median values of fluorescence intensity histograms as previously described [21].

### Survival in acidic microenvironment

Primary and metastatic melanoma cell lines were seeded in RPMI 1640 with 10% FCS at pH values of 7.4 or 5.5, as previously described. The pH value of 5.5 was obtained by adding 2 N HCl to the RPMI 1640 culture medium [21]. After 5 day in culture, cells were stained with 0.4% Trypan blue and analyzed by flow cytometry.

### Expression of cathepsins B, D and L and cystatin C

Total amount of cathepsin B and D and L were evaluated by Western blot as previously reported [22]. Cell surface cathepsin B and D and L were detected by flow cytometry in unfixed living cells stained with specific primary antibodies (for cathepsin B and D, polyclonal antibody Calbiochem; for cathepsin L, monoclonal antibody, Alexis, Lausen, Switzerland) for 45 min on ice. After washings, cells were incubated for additional 30 min with a secondary Alexa-488-conjugated anti-rabbit or anti-mouse antibody (Molecular Probes, Eugene, OR, USA). Cells were analyzed on a cytometer or, alternatively, were fixed for 10 min in 4% paraformaldehyde and observed with a Nikon Microphot fluorescence microscope. Images were captured by a color chilled 3 charge-coupled device (CCD) camera (Hamamatsu, Japan) and analyzed by the OPTILAB (Graftek, France) software. For evaluation of cystatin C expression level, fixed cells were permeabilized and then stained with specific monoclonal antibody against cystatin C (10 μg/ml, Abcam Ltd.21 Cambridge, UK) as previously reported for other intracellular antigens [22]. Samples were analyzed on a FACScan flow cytometer (Becton Dickinson, San Jose', CA, USA) by using FL-1 detector.

### Assay for activity of cathepsins

Cathepsin B and D and L activity in the cell medium were evaluated by using sensitive fluorogenic substrates: Abz-Gly-Ile-Val-Arg~Ala-Lys(Dnp)-OH (Ex: 320; Em: 420), D Bz-Arg-Gly-Phe-Phe-Pro-4MeObNA, HCl (Ex: 345; Em: 425) and Ac-His-Arg-Tyr-Arg-ACC (Ex:380; Em: 460) specific for cathepsin B, D and L, respectively (all Calbiochem) as previously reported [23]. Fluorescence of the samples (in triplicate for each experimental condition) was read by using a microplate fluorometer.

### Cystatin C and VEGF-A evaluation in the culture medium

Evaluation of cystatin C and vascular endothelial growth factor A (VEGF-A) in cell culture medium was performed with specific ELISA kits from Alexis and Bender MedSystem (Lausen, Switzerland), respectively. According to the manufacturer instructions, medium samples were analyzed by a plate microreader with 450 ± 10 nm filter. Detected values were compared with a standard curve and reported as ng/ml (cystatin C) or pg/ml (VEGF-A).

### Cell invasion assays

Tumor cell invasion was determined *in vitro *by using transwell culture inserts (8.0-μm pore size) coated with Matrigel (Becton Dickinson) as previously reported [24]. Each assay was carried out at least three times in triplicate for each experimental condition. To determine cathepsin B surface amount, cells on the filter top or migrating to the bottom surface of the filter were harvested and stained as reported above.

### Cathepsin B gene silencing with siRNA

Melanoma cells were cultured in antibiotic-free medium and transfected with Dharma FECT 4 reagent (Dharmacon, Lafayette, CO), according to the manufacturer's instructions, using 100 nM siRNA human cathepsin B, D and L. The transfection efficiency was confirmed by using a Dharmacon's positive silencing control, siGLO laminin A/C siRNA. After 24 h, the culture medium was replaced with fresh medium and transfected again, as above, with 100 nM siRNA. The siRNAs targeting human cathepsins were the following. For cathepsin B: r(GGAUCACUGCGGAAUCGAA)dTdT and r(UUCGAUUCCGCAGUGAUCC)dTdG; for cathepsin D: r(AAGUGGUGGACCAGAACAUC)dTdG and for cathepsin L: 5'-GGCGATGCACAACAGATTATT-3'. After further 48 h, the effect of transfection on protein expression was verified by Western blot or flow cytometry analyses. Cells knocked down for cathepsins were then tested for their invasion capability.

### In vivo experiments

CD-1 male nude (nu/nu) mice, 6-8 weeks old and weighing 22-24 g were purchased from Charles River Laboratories (Calco, Italy). All procedures involving animals and their care were in accordance with institutional guidelines under the control of the Italian Ministry of Public Health (guided by International Guideline Principles for Biomedical Research Involving Animals developed by the Council for International Organization of Medical Sciences). To study the effect of CA-074 on the growth of MM1 human melanoma, cells in exponential phase of *in vitro *growth were injected into the hind leg muscles of mice at 5 × 10^6 ^cells/mouse. Mice were treated as from day 6 after tumor implant when a tumor mass of about 250 mg was evident, by injecting CA-074 i.v. at 10 mg/kg for eight consecutive days. This dose was chosen based on previous results [25] and on our preliminary experiments showing the tolerability of the treatment on healthy mice. Control animals received vehicle alone. Tumor weight was calculated by caliper measurements as previously reported [26]. Antitumor efficacy of treatments was assessed by the following end-points: a) percent tumor weight inhibition, calculated as [1-(mean tumor weight of treated mice/mean tumor weight of controls)] x 100; b) tumor growth delay, evaluated comparing the median times for treated and untreated tumors, respectively, to achieve equivalent size. To evaluate the antimetastatic efficacy of CA-074, MM4 cells were injected i.v. at 5 × 10^4 ^cells/mouse. The day after treatment the drug was administered i.v. by using the same schedule reported above. Control animals were injected with vehicle alone. Two weeks after the injection of cells, mice were killed, their lungs removed and fixed in Bouin's solution to distinguish tumor nodules from lung tissue, and the number of metastases was determined under a stereomicroscope. The efficacy of treatment was assessed by comparing the reduction in the number of metastases in treated versus untreated mice. Each experimental group consisted of six mice.

### Data analysis and statistics

All samples were analyzed with a FACScan cytometer (Becton Dickinson) equipped with a 488 argon laser. At least 20,000 events were acquired. The expression level of the analyzed proteins was expressed as a median value of the fluorescence emission curve. Statistical significance among different experimental conditions of the same experiment was calculated by using the parametric Kolmogorov-Smirnov (K/S) test. Statistical analysis among different experiments was performed by Student's *t*-test by using Statview program for Macintosh. All data reported were verified at least in three independent experiments and expressed as mean ± standard deviation (SD). Only *p *values of less than 0.01 were considered as statistically significant.

## Results

### Expression of cathepsins in cells from primary and metastatic human melanomas

**Figure 1:**
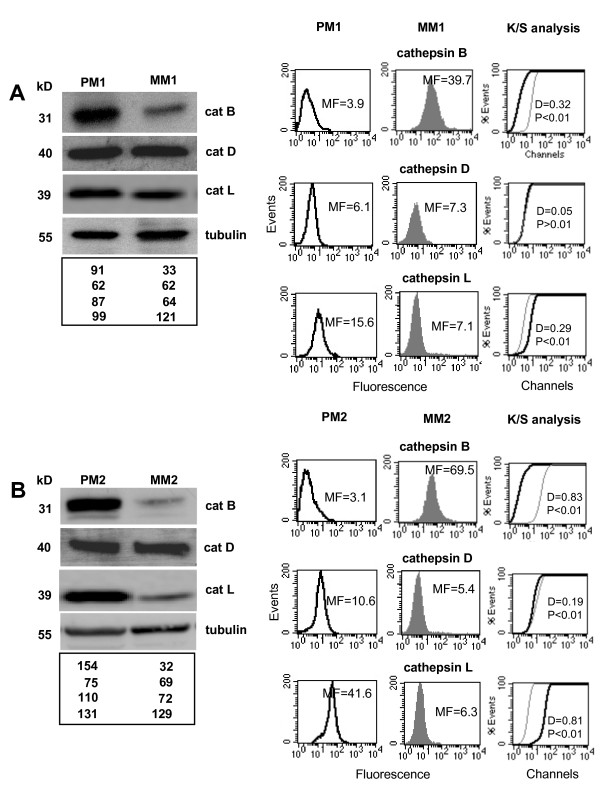
**Cathepsin B expression in cells from primary and metastatic human melanomas**. Western blot analysis (left panels) of cathepsin B, cathepsin D, cathepsin L and tubulin in the primary melanoma cell lines PM1 (**A**) and PM2 (**B) **and in the metastatic melanoma cell lines MM1 (**A**) and MM2 (**B**). Quantitative flow cytometry analysis (right panels) of plasma membrane cathepsin B in PM1 (**A**) and PM2 (**B**) cell lines and in MM1 (**A**) and MM2 (**B**) cell lines. The values of Western blot signals, reported in the bottom panels of (**A**) and (**B**), were obtained by densitometric analysis and expressed as arbitrary units (a.u.). Numbers in the right panels of (**A**) and (**B**) represent the median fluorescence values. In the right panels of (**A**) and (**B**) statistical analysis performed by non-parametric K/S test is reported.

**Figure 2:**
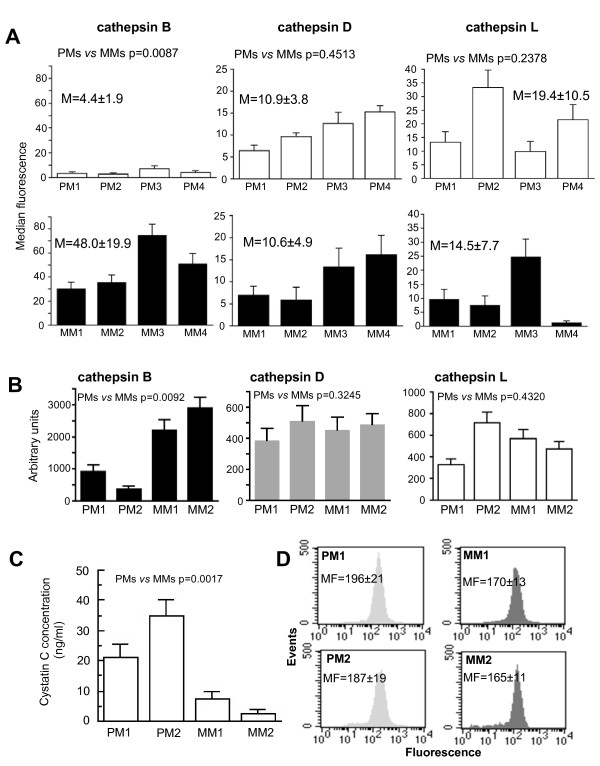
**Cathepsin activity and cystatin C amount in primary and metastatic human melanoma cells**. (**A**) Quantitative cytofluorimetric analysis of plasma membrane cathepsin B, D and L in four different primary (white columns) and metastatic (black columns) melanoma cell lines. Numbers represent the mean values among four different primary or metastatic melanoma cell lines analyzed. (**B**) Fluorimetric assays for cathepsin B (black columns), cathepsin D (grey columns) and cathepsin L (white columns) activity in the cell medium of two representative primary and metastatic melanoma cell lines. Results are reported as fluorescence units. (**C**) Concentration of cystatin C in the growth medium of two representative primary and metastatic melanoma cell lines obtained by ELISA test. Results are reported as ng/ml. (*) *p *< 0.01 for PM cell lines *vs. *MM cell lines by Student's *t*-test. (**D**) Quantitative cytofluorimetric analysis of cystatin C intracellular content in two representative primary (light grey histograms) and metastatic (deep grey histograms) melanoma cell lines. Numbers represent the median fluorescence values ± SD among four independent measurements. Histograms from a representative experiment are shown.

We analyzed the total amount of cathepsin B, D and L in four cell lines derived from human metastatic melanomas (MM1-MM4) and in four cell lines derived from primary lesions (PM1-PM4) by means of Western blotting. It appeared evident that the amount of cathepsin B resulted higher in cells from primary melanomas rather than in those from metastatic ones. This is shown in Figure 1A and 1B (in which two representative primary and two metastatic melanoma cell lines are shown) by the values obtained by densitometric analysis of the relative bands (bottom panels). As far as cathepsin D was concerned, we found comparable amounts in cells derived from primary or metastatic melanomas, whereas the expression level of cathepsin L was variable and not associated with malignancy. Conversely, the evaluation of surface expression of cathepsins by flow cytometry revealed that cathepsin B was higher in cell lines from metastatic melanomas (MM1 and MM2 as representative cell lines) (Figures 1A and 1B, right panels) than in cell lines derived from primary melanomas (PM1 and PM2 as representative cell lines). These data were also confirmed by extending the cytofluorimetric analysis to a total of four different primary (PM1-PM4) and four metastatic (MM1-MM4) melanoma cell lines (Figure 2A). In fact, a significantly higher expression of cathepsin B at the cell surface of metastatic melanoma cell lines (left panels, *p *= 0.0087) was observed. By contrast, both for cathepsin D and cathepsin L surface expression, statistical analysis failed to reveal any significant difference between primary and metastatic melanoma cells (*p *= 0.4513 and *p *= 0.2378, respectively), at least considering these eight cell lines.

The above results obtained with cathepsin B seem in conflict with some literature data showing higher amounts of cathepsin B mRNA or protein in malignant tumors than in normal tissues or benign tumors [4,27]. On these bases, we performed, by quantitative real-time RT-PCR, cathepsin B specific mRNA determination. Being this protease a post-translational derivative of the enzymatically inactive procathepsin B, we analyzed the primary PM1 and the metastatic MM1 cell lines and the autologous cell lines, PM2, from a primary human melanoma, and MM2, from a supraclavicular lymph node metastasis, for procathepsin B mRNA content. The results, reported in Additional File 1, showed a significantly higher content of procathepsin B transcripts in the MM1 cell line, when compared to PM1 (+ 58%) and in MM2 cell line *versus *PM2 cell line (+ 75%).

### Cathepsin B activity in primary and metastatic human melanoma cells

As clearly shown in Figure 2B (left panel), where results obtained in two representative primary or metastatic melanoma cell lines are shown, cathepsin B activity was significantly higher in the growth medium of metastatic than that of primary melanoma cells (*p *< 0.01). By contrast, no significant differences in its activity were found using specific fluorescent substrates for cathepsin D (Figure 2B, central panel) or cathepsin L (Figure 2B, right panel). Furthermore, the concentration of cystatin C, an endogenous cathepsin B inhibitor, was found higher in the growth medium of PM1 and PM2 in comparison with MM1 and MM2 cells (Figure 2C, p = 0.0017). As far as the intracellular content of cystatin C was concerned (Figure 2D), flow cytometry analysis revealed a modest, but significantly (*p *< 0.01) higher amount of this physiological cathepsin B inhibitor in cell lines from primary melanoma in comparison to those from metastatic lesions. In Figure 2D two representative cell lines from primary or metastatic melanoma lesions are shown.

### Analysis of the lysosomal compartment

We analyzed acidity and volume of the lysosomal compartment in four different melanoma cell lines both from primary and metastatic lesions (Additional File 2A and 2B). The results showed that the intracytoplasmic vesicles were localized in invadopodia, as previously described [28] (data not shown) and were significantly more acidic in cells from metastatic melanomas with respect to primary melanomas (*p *= 0.0083, Additional File 2A, left and right panels, respectively). By contrast, no significant difference was found in terms of lysosomal volume (*p *= 0.2187, bottom panels in Additional File 2B, left and right panels, respectively). We also found that cells from metastatic melanomas (Additional File 2C, right panel) were able to survive at low pH values (5.5), whereas cells from primary lesions (left panel) rapidly died when cultured under the same conditions (primary *vs. *metastatic cells, *p *= 2.2 × 10^-7^). These data are potentially interesting in this contest because it has been reported that acidic pH can promote cancer dissemination by a mechanism involving acidity-induced up-regulation of some proteolytic enzymes such as metalloproteinase-2, cathepsin B, cathepsin L and the pro-angiogenic factors VEGF-A and IL-8 [29].

### Cathepsin B and in vitro invasiveness: effects of chemical and biological inhibitors

**Figure 3:**
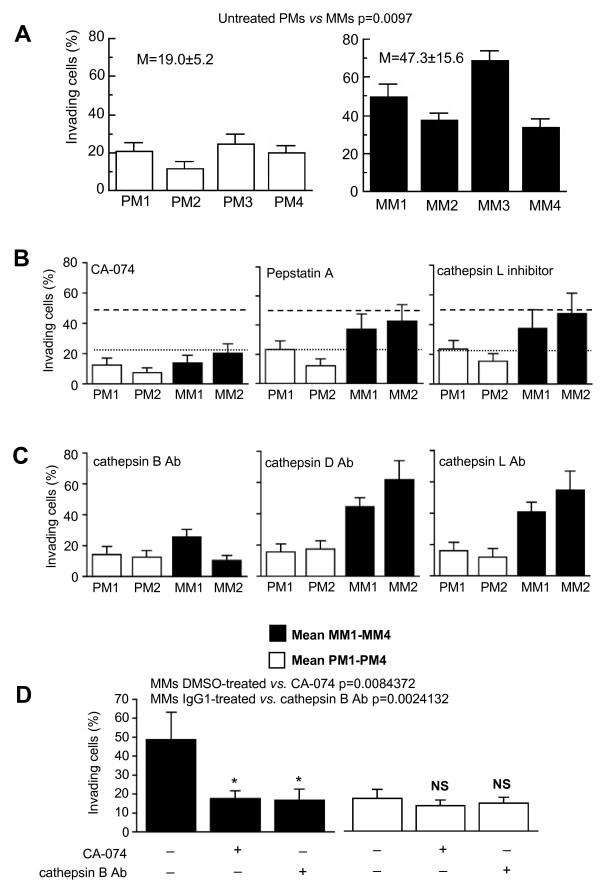
***Cathepsins and in vitro invasiveness: *effect of chemical and biological inhibitors**. (**A**) Invasion test on primary (white columns) and metastatic (black columns) melanoma cell lines. (**B**) Invasion test on two representative cell lines from primary (white columns) and metastatic (black columns) melanoma in the presence of CA-074 (left panel), Pepstatin A (central panel) or cathepsin L inhibitor (right panel). Dotted lines represent the mean of the results obtained in primary melanoma cell lines, and dashed lines indicate the mean of the results obtained in metastatic melanoma cell lines by using DMSO (vehicle of the cathepsin inhibitors). (**C**) Invasion test on two representative cell lines from primary (white columns) and metastatic (black columns) melanoma in the absence or in the presence of specific antibodies against cathepsin B (left panel), cathepsin D (central panel) or cathepsin L (right panel). Dotted lines represent the mean of the results obtained in primary melanoma cell lines, and dashed lines indicate the mean of the results obtained in metastatic melanoma cell lines by using IgG1 as positive control. (**D**) Histogram showing the results obtained from four different primary melanoma (white columns) or metastatic melanoma (black columns) cell lines in the absence or in the presence of CA-074 or antibodies against cathepsin B. Data are reported as mean ± SD of the percentage of invading cells. Student's *t*-test indicates: *p *= 0.0097 for untreated MM cells *vs. *CA-074-treated MM cells and *p *= 0.0030 for untreated MM cells *vs. *MM cells treated with anti-cathepsin B antibodies.

The relationship between *in vitro *invasiveness and cathepsin B localization at the plasma membrane was then analyzed in four different cell lines from both primary and metastatic human melanomas (Figure 3). All the cell lines from metastatic melanomas showed a significantly higher ability to cross through Matrigel than those from primary lesions (Figure 3A, *p *= 0.0097). Synthetic cysteinyl proteinase cathepsin B inhibitor CA-074, but not synthetic aspartyl proteinase cathepsin D inhibitor Pepstatin A and cathepsin L inhibitor, significantly reduced the percentage of invading cells (Figure 3B, two representative cell lines from primary and metastatic melanoma are shown). In the same vein, we observed that the presence of specific antibodies to cathepsin B in the culture medium significantly decreased the percentage of metastatic melanoma cells able to cross through a basement membrane matrix, i.e. Matrigel (Figure 3C, left panel). According to the results reported in Figure 3B, the presence of specific antibodies to cathepsin D or cathepsin L in the culture medium did not significantly reduce the percentage of cells migrating to the bottom surface of the filter (Figure 3C, central and right panel, respectively). Thus, on the bases of statistical analyses of the data obtained in four different primary (PM1-PM4) and metastatic (MM1-MM4) melanoma cell lines, we can conclude that: i) inhibitors of cathepsin D and L, as well as specific antibodies against both these proteases, were ineffective in reducing invasion capability of both primary and metastatic melanoma cell lines and that ii) either the chemical inhibitor of cathepsin B, CA-074, or specific anti-cathepsin B antibodies were able to significantly prevent the *in vitro *invasiveness of metastatic melanoma cell lines (Figure 3D).

### Cathepsin B surface expression and in vitro invasiveness

**Figure 4:**
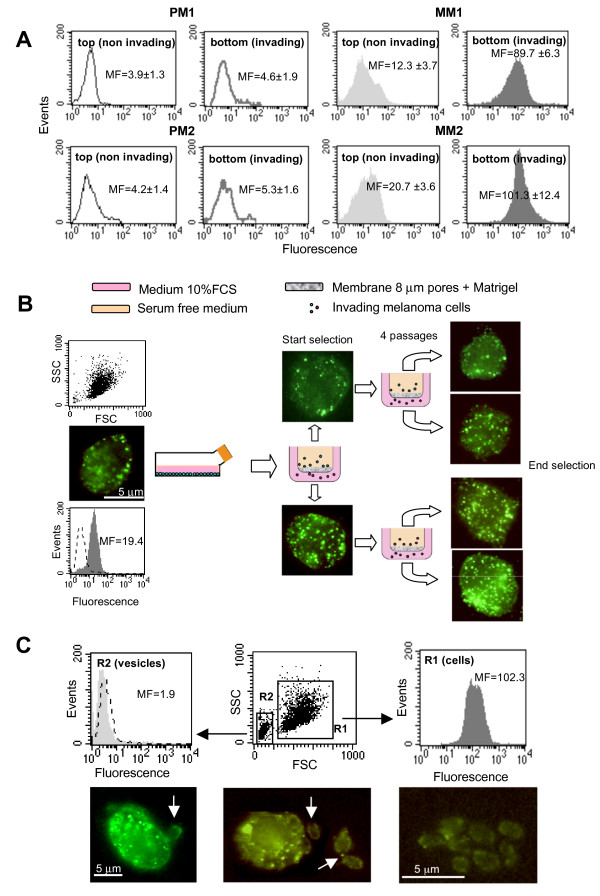
**Cathepsin B surface expression and in vitro invasiveness**. (**A**) Quantitative flow cytometry analysis of plasma membrane cathepsin B in PM1 and PM2 cell lines (empty histograms) and in MM1 and MM2 cell lines (full histograms) performed in cells found above (non invading) or below (invading) the Matrigel covered filter. Numbers represent the median fluorescence values ± SD among three independent measurements. Histograms from a representative experiment are shown. (**B**) Cytofluorimetric and fluorescence microscopy analyses of plasma membrane cathepsin B in MM4 cell line during selection of invading cells by repeated passages through the Matrigel-covered filters. (**C**) Cytofluorimetric analysis of plasma membrane cathepsin B in MM4 cells at the end of the selection after four passages through the Matrigel-covered filters revealed the presence of a sub-population with reduced size (R2 region, bottom central panel) and negative to cathepsin B (bottom left panel). In the bottom panels fluorescence microscopy micrographs showing cathepsin B-positive cells (corresponding to R1 region) and cathepsin B-negative microvesicles, probably corresponding to the sub-population (R2 region) revealed by cytofluorimetric analysis. Arrows indicate vesicles budding from (left picture) or near to (central picture) the cell. In the right panel a cluster of vesicles is shown.

Differential analysis of cathepsin B surface expression performed in cells found above (non-invading) or below (invading) the Matrigel-covered filter, clearly demonstrated that invading cells (either from primary or metastatic melanomas) showed a significantly higher amount of cathepsin B at the cell surface (*p *< 0.01) (results obtained in two representative cell lines from primary and metastatic melanoma lesions are shown in Figure 4A).

Interestingly, by selecting invading cells by repeated passages through the Matrigel-covered filters, as illustrated in Figure 4B, we observed a progressive increase of surface cathepsin B amount. In particular, we selected MM4 cell line by four passages through the filter, seeding separately at any passage on distinct transwell culture inserts the cells found above or those migrating below the filter. Flow cytometry analysis of physical parameters of these cells (after selection) also revealed the appearance of a lower-sized cell sub-population (Figure 4C, R2 in central bottom panel), essentially negative for surface cathepsin B determination (Figure 4C, left bottom panel). Immunofluorescence microscopy analysis confirmed the presence of several cathepsin B-negative vesicles (Figure 4C, arrows in lower panels) possibly budding from the cell surface (Figure 4B, arrow in the lower left panel). The nature and biological significance of these vesicles are not clear and should deserve more exhaustive studies.

### Cathepsins and in vitro invasiveness: effect of siRNA

**Figure 5:**
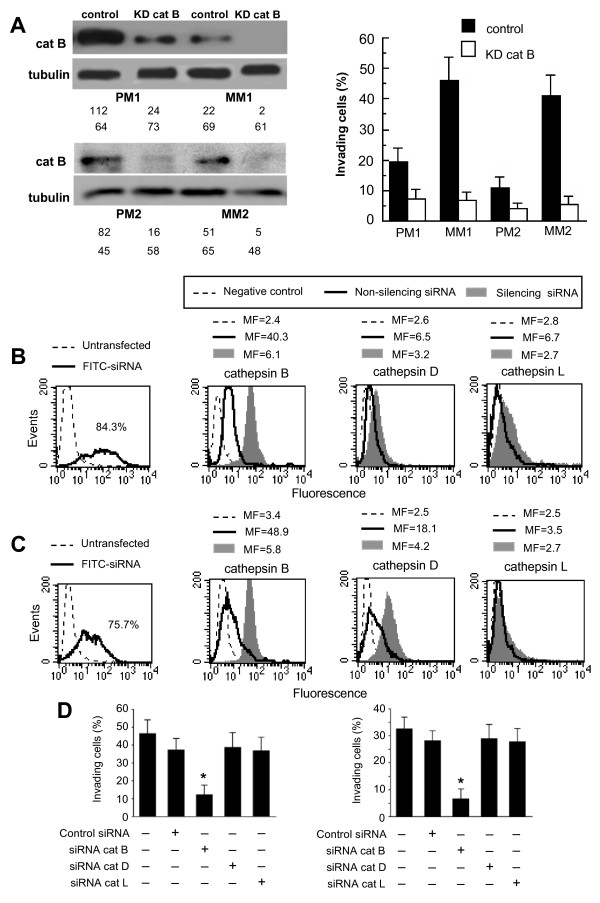
***Cathepsins and in vitro invasiveness*: effect of siRNA**. (**A**) Western blot analysis of cathepsin B (left panels) and invasion test (right panel) of cells knocked down for cathepsin B in two different primary (PM1 and PM2) and metastatic (MM1 and MM2) melanoma cell lines. The values of Western blot signals, reported in the bottom left panels, were obtained by densitometric analysis and are expressed as arbitrary units (a.u.). *In vitro *invasion test (right panel) in cells knocked down for cathepsin B showed a significant (*p *< 0.01) reduction of their invasion capability. Data are reported as mean ± SD of the percentage of invading cells obtained in three independent experiments. Two representative primary or metastatic melanoma cell lines are shown. (**B **and **C**) Flow cytometry evaluation of fluorescence in MM1 cells (**B**) or MM4 cells (**C**) transfected with FITC-siRNA. The number in the left panel represents the percentage of FITC-positive cells (corresponding to transfected cells). Cytofluorimetric evaluation, performed 48 h after transfection, of cathepsin B (second panel), D (third panel) or L (fourth panel) in MM1 (**B**) and MM4 (**C**) cells transfected with non-silencing siRNA or with silencing siRNA. Numbers represent the median fluorescence intensity and indicate the expression level of the cathepsins. A representative experiment among three is shown. (**D**) Invasion test of cells knocked down for cathepsins in MM1 (left panel) and in MM4 (right panel) cell lines. (*) Indicates *p *< 0.01.

Finally, according to the above results, cells knocked-down for cathepsin B (Figure 5A, left panel) displayed a significant (*p *< 0.01) reduction of their invasiveness (Figure 5A, right panel). This was clearly evident in cell lines derived from primary melanomas (PM1 and PM2) and in those derived from metastatic lesions (MM1 and MM2) as well.

On the bases of these results, we decided to contextually transfect either MM1 (Figure 5B) or MM4 (Figure 5C) cells, the two metastatic melanoma cell lines tested in *in vivo *experiments, with siRNA silencing cathepsin B, D and L. Forty-eight hours after transfection, we evaluated by flow cytometry: (i) the percentage of transfected cells (Figure 5B, MM1 cell line and Figure 5C, MM4 cell line) and (ii) the expression level of cathepsin B (second panel) cathepsin D (third panel) and cathepsin L (fourth panel). Knocked down cells were then tested for their invasion capability. We found that cathepsin B silencing significantly reduced invasion capability in both MM1 (Figure 5D, left panel) and MM4 (Figure 5D, right panel) cell line. By contrast, neither cathepsin D nor cathepsin L silencing significantly affected the invasiveness of these two metastatic cell lines.

### In vivo efficacy of CA-074 on human melanoma xenografts

**Figure 6:**
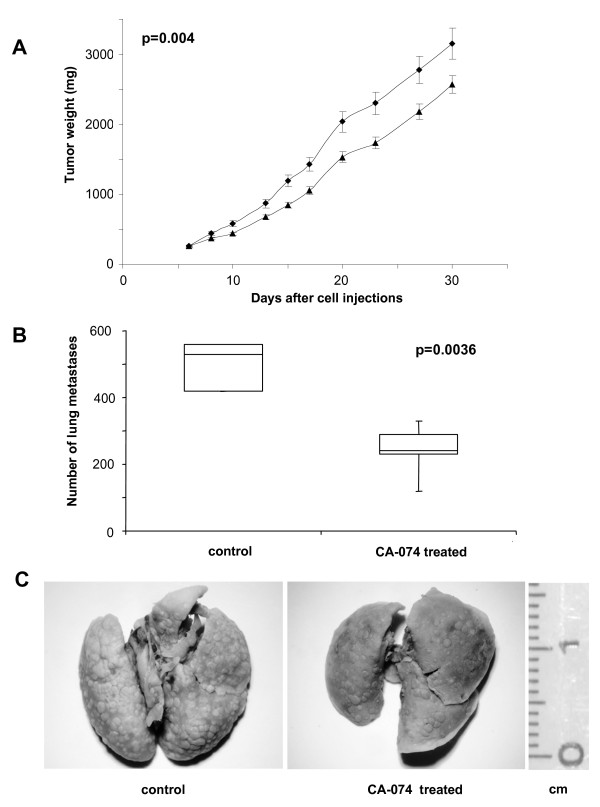
**Antitumor efficacy of CA-074 in human melanoma xenografts**. (**A**) Tumor growth curves of mice bearing MM1 melanoma cells and treated i.v. with vehicle alone (υ) or with CA-074 (σ) for eight consecutive days, starting from day 6 after cell injection. Mean tumor weights in mg ± SD are shown. (**B**) Mice were injected i.v. with MM4 cells and the day after, treated with CA-074 for eight consecutive days. At day 14 after cells injection, lungs were removed and fixed in Bouin's solution to distinguish tumor nodules from lung tissue, and the number of metastases was evaluated. The boxes show medians and 75th and 25th percentiles of the data, while the whiskers indicate the minimum and maximum values. (**C**) Representative images of metastatic nodules in lungs from control and treated mice. Note the lower number of nodules in the right picture.

The MM1 the highly metastatic MM4 human melanoma cells were used to investigate the antitumor activity of CA-074 administered systemically in mice. Indeed, the treatment of human melanoma-bearing mice significantly reduced the growth of i.m.-injected tumors and inhibited the number of artificial lung metastases. As shown in Figure 6A, CA-074 injected in MM1 xenografts produced about 30% inhibition of tumor weights when compared to control mice (*p *= 0.00025), as evaluated at nadir of the effect. Such inhibition was accompanied by a tumor growth delay of 7 days, significantly different (*p *= 0.004) when compared to mice treated with the vehicle alone (dimethyl sulfoxide, DMSO).

With the purpose to analyze the effect of CA-074 on the metastatic potential of melanoma cells, mice were injected i.v. with MM4 cells, a very aggressive human melanoma cell line [26], being MM1 cells unable to induce artificial lung metastases when injected i.v. Interestingly, MM4 cells produced and secreted more than 50-fold VEGF-A (226.6 ± 31.3 pg/ml) when compared to MM1 cells (4.73 ± 1.9 pg/ml), a feature likely associated with the high aggressiveness shown by the MM4 cell line. Extending this analysis to all cell lines considered here, we also found that the amount of VEGF-A in the growth medium of the metastatic melanoma cells (49.2 ± 83.7 pg/ml) resulted significantly higher (*p *= 2.9 × 10^-11^) than that found in the primary melanoma cells (0.5 ± 0.2 pg/ml). As far as the metastatic potential is concerned, we found that mice treated with vehicle alone showed a median number of 530 lung nodules (range 310-600). Treatment with CA-074 significantly reduced the number of nodules (median number 240, range 178-310) (*p *= 0.0036) (Figure 6B). Representative images of lungs removed from control or CA-074 treated mice are reported in Figure 6C.

## Discussion

In the present work we investigated the role of cathepsin B, D and L in metastatic melanoma aggressiveness. Unlike cathepsins D and L, cathepsin B appears to contribute significantly to cell spreading and metastatic potential. We also show for the first time that the specific cathepsin B inhibitor CA-074, as well as antibodies directed against cathepsin B, was able *per se*, i.e. without any further additional drug, to exert a powerful anti-invasive activity by a mechanism that brings into play the impairment of metastatic cell dissemination. This was assessed both *in vitro*, i.e. in eight different cell lines from primary and metastatic lesions, as well as *in vivo*, i.e. in murine xenografts, where a decrease of tumor weight and an increased tumor growth delay as well as a significant reduction of artificial lung metastases were detected after CA-074 administration.

The possible link between cathepsins and cancer and the role of cathepsin B in metastatic potential was postulated since many years [30,31]. For instance, numerous clinical studies have hypothesized a correlation between extralysosomal cathepsin B expression and release with neoplastic disease progression and clinical outcome [5]. However, in spite of the number of papers published in the field, the mechanisms and the pathways involved are still under investigation. Cathepsin B, either the mRNA or the protein, was often detected in higher amount in malignant tumors than in benign ones or in normal tissues [27]. In addition, the intracellular trafficking of cathepsin B appeared frequently altered in malignant tumors [32], resulting in i) an increased secretion of precursor and active forms of the enzyme [33], ii) its redistribution from perinuclear lysosomes to peripheral vesicles localized in invadopodes [34], and finally iii) its association with the plasma membrane [35], where it was found associated to the caveolae, via active K-RAS, at least in colon cancer [36]. Some investigators hypothesized a role for cathepsin B in the mechanisms of invasion and metastasis [4]. In our experimental model, in spite of the higher amount of cathepsin B mRNA in metastatic melanoma cell lines in comparison to primary melanoma cell lines, we observed that protein cathepsin B content was higher in primary melanoma than in metastatic melanoma cell lines. Thus, we can hypothesize that this discrepancy could be due to a major secretion of cathepsin B by metastatic cells. According to this, our analyses performed at different time points in invading cells after selections by repeated passages through the Matrigel-covered filters, suggested that plasma membrane localization of cathepsin B and its extracellular activity, rather than its overall expression, had a major correlation with cell invasion capabilities.

The pivotal and specific role of cathepsin B in metastatic melanoma cells was also reinforced by the fact that: i) differently from cathepsin B, the activity and the expression of other two cathepsins analyzed here, cathepsin D and cathepsin L, were found essentially unchanged in metastatic cells with respect to primary melanoma cells. Furthermore, ii) specific inhibitors of cathepsin D and cathepsin L, i.e. pepstatin A and cathepsin L inhibitor II, respectively, as well as their specific siRNA, did not modify metastatic cell invasiveness *in vitro*. Accordingly, cathepsin D was reported to be involved in cellular transformation, being down-regulated in melanoma cells with respect to melanocytes [37], rather than in metastatic potential. As concerns cathepsin L, this belongs to the cysteine protease family and its expression was suggested to correlate with increased invasion ability of tumor cells, e.g. of M14 murine melanoma cells *in vitro *[38]. At variance, we did not find any significant association between the expression of this cathepsin and invasion capability. This seems in accord with the hypothesis that high cathepsin L concentrations are detectable in primary melanomas with a poor prognosis [39].

As concerns cystatins, including cystatin C, they function as cysteine protease inhibitors. These are expressed in numerous cell types and regulate a number of biological processes, including tumor progression. Cystatins are epigenetically silenced through DNA methylation-dependent mechanisms in several forms of cancer and they have been hypothesized to regulate promotion or suppression of tumor growth, invasion, and metastasis [40]. Our results indicated that high levels of cystatin C could only be detected in the milieu of melanoma cells from primary lesions. Hence, it cannot be excluded that the low level of this physiological cathepsin inhibitor found in the growth medium of metastatic melanoma could be the result of its "exhaustion", e.g. consequent to the attempt to counteract cathepsin activity.

As a general rule, cathepsins are optimally active at an acidic pH and extracellular pH is lower in many tumors rather than in the corresponding normal tissue microenvironment. Cells cultured at acidic pH have been reported to increase the secretion of proteinases and proangiogenic factors and to enhance invasive and angiogenic potential as well as the potential to develop experimental metastases [29]. Our data indicated that, conversely to cells from primary melanoma, metastatic cells were able to survive at acidic pH (see Supplementary Figure 1), also supporting the evidence that more malignant tumors are able to grow and expand in an acidic environment, as that ones produced under hypoxic conditions [41]. Thus, a reappraisal of treatment strategies involving deliberate tumor acidification to improve the efficacy of chemotherapy should be considered. The possibility that cathepsins, including cathepsin B, could play a key role in the formation of acidic tumor microenvironment should also be taken into account. A further point in this context concerns VEGF secretion. It was suggested that acidic pH promotes experimental metastasis by human melanoma cells via a mechanism involving acidity-induced up-regulation of proteolytic enzymes, including cathepsins, as well as up-regulating pro-angiogenic factors such as VEGF [29]. Hence, considering their high production of VEGF, the aggressive behavior of MM4 cells, once injected in mice, it is not surprising and the effects obtained with CA-074 may also be partially referred to as its putative microenvironmental "buffering activity".

The last important point regards the fact that either chemical inhibitors, i.e. CA-074 and CA-074Me, or biological, i.e. antibodies directed against active cathepsin B, as well as cathepsin B gene silencing with siRNA, determine a significant impairment of cell aggressiveness in terms of spreading ability. The block of cathepsin B activity via antibody administration, unlike that of chemical compounds and siRNA, could impair metastatic melanoma cell dissemination certainly exerting their activity at extracellular level. Since the use of immunotherapeutic strategies recently acquired a clinical relevance, specific *in vivo *studies with antibodies against cathepsin B would be planned.

In conclusion, our paper suggests a role for cathepsin B in tumor growth and in metastatic potential of human melanoma. Inhibition of cathepsin B could possibly lead to changes of tumor microenvironment, to a decreased cell survival and spreading and, therefore, to the impairment of metastatic seeding and onset. Further research on melanoma, as well as on other tumor models, will however be crucial to clarify these points before generating therapeutic strategies based on the use of available or novel potential inhibitors of cathepsin B activity.

## Abbreviations

CA-074: L-trans-epoxisuccinil-lle-Pro-OH propylamide; CA-074 Me: L-trans-epoxisuccinil-lle-Pro-OH propylamide methyl ester; CCD camera: charge-coupled device camera; DMSO: dimethyl sulfoxide; FCS: fetal calf serum; I.V.: intra venous; K/S: Kolmogorov-Smirnov test; MM: metastatic melanoma cell line; PM: primary melanoma cell line; PepA: Pepstatin A; SD: standard deviation; siRNA: small interference RNA; VEGF-A: vascular endothelial growth factor A

## Competing interests

The authors declare that they have no competing interests.

## Authors' contributions

PM participated in designing the study, designed all the experiments, participated in carrying out the cell biology studies, analyzed and interpreted the data, supervised other co-authors, participated in performing the statistical analysis, wrote the manuscript; BA, LC, AMM, and RV participated in carrying out biochemical and molecular studies, analyzed and interpreted data; CL and MS designed all the in vivo experiments, analyzed and interpreted the in vivo data, and participated in performing the statistical analysis; CC participated in designing the study, furnished clinical expertise and biological materials (fresh isolated melanoma cells), analyzed and interpreted data; WM and MGP participated in designing the study, analyzed and interpreted the data, supervised other co-authors performing biochemical and molecular studies, participated in performing the statistical analysis, participated in writing the manuscript.

All the author read and approved the final manuscript

## Supplementary Material

Additional file 1 **Relative quantification of procathepsin B gene expression in primary and metastatic melanoma cells by real-time quantitative PCR**. The data indicate an increase in procathepsin B gene expression in the metastatic MM1 and MM2 cell lines, when compared with the primary melanomas PM1 and PM2, respectively. Values, average of two experiments performed in triplicate ± SD, are normalized against cyclophilin A. TAI, transcription activation index for procathepsin B. Click here for file

Additional file 2 **Analysis of the lysosomal compartment and cell survival in acidic environment**. Cytofluorimetric evaluation of lysosomal acidity (**A**) and lysosomal volume (**B**) in four different primary (white columns) and metastatic (black columns) human melanoma cell lines by using LysoSensor-green and LysoTracker-green dye, respectively. Data are reported as mean ± SD of the median fluorescence values among four independent experiments. Statistical analysis by Student's *t*-test indicates: *p *= 0.0083 for lysosomal acidity and *p *= 0.1287 for lysosomal volume for PM cell lines *vs. *MM cell lines. (**C**) Cell survival analysis at different pH values of the growth medium was performed by Trypan blue test. Data are reported as mean ± SD of the percentage of surviving cells obtained in three separate experiments performed in triplicate. Statistical analysis by Student's *t*-test indicates: *p *= 2.2 × 10^-7 ^for PM cell lines *vs. *MM cell lines at pH 5.5. Click here for file
