# Supplementary material for: Cathepsin B inhibition interferes with metastatic potential of human melanoma: an in vitro and in vivo study
Source: Mol Cancer. 2010 Aug 4;9:207. doi: 10.1186/1476-4598-9-207 (PMC2925371; doi:10.1186/1476-4598-9-207)

Relative quantification of procathepsin B gene expression in primary and metastatic melanoma cells by real-time quantitative PCR.

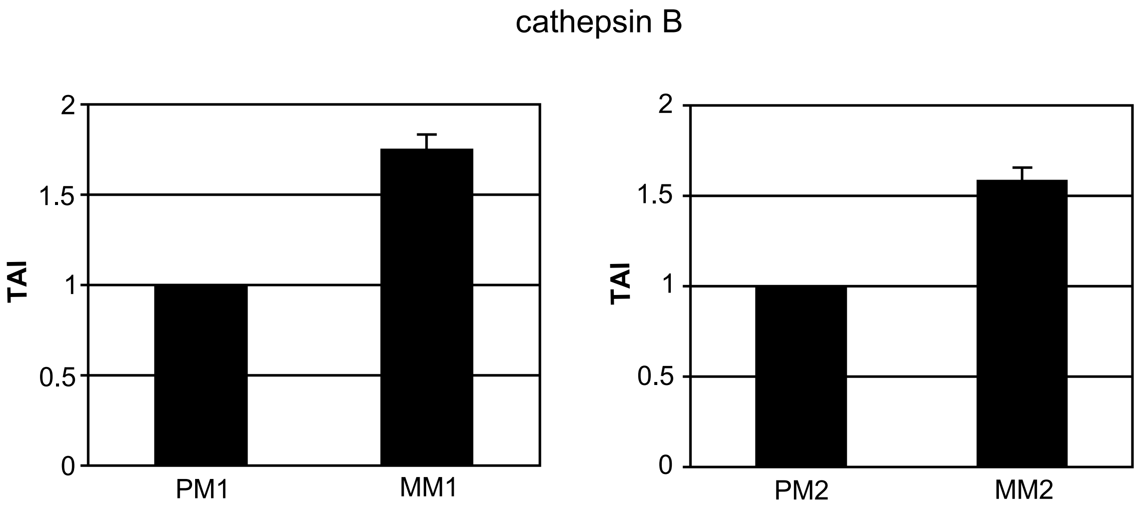

Supplement: Additional file 1 — Relative quantification of procathepsin B gene expression in primary and metastatic melanoma cells by real-time quantitative PCR. The data indicate an increase in procathepsin B gene expression in the metastatic MM1 and MM2 cell lines, when compared with the primary melanomas PM1 and PM2, respectively. Values, average of two experiments performed in triplicate ± SD, are normalized against cyclophilin A. TAI, transcription activation index for procathepsin B. [file 1476-4598-9-207-S1.PDF]
