# Supplementary figures and images for: Cathepsin B inhibition interferes with metastatic potential of human melanoma: an in vitro and in vivo study
Source: Mol Cancer. 2010 Aug 4;9:207. doi: 10.1186/1476-4598-9-207 (PMC2925371; doi:10.1186/1476-4598-9-207)

## Analysis of lysosomal compartment

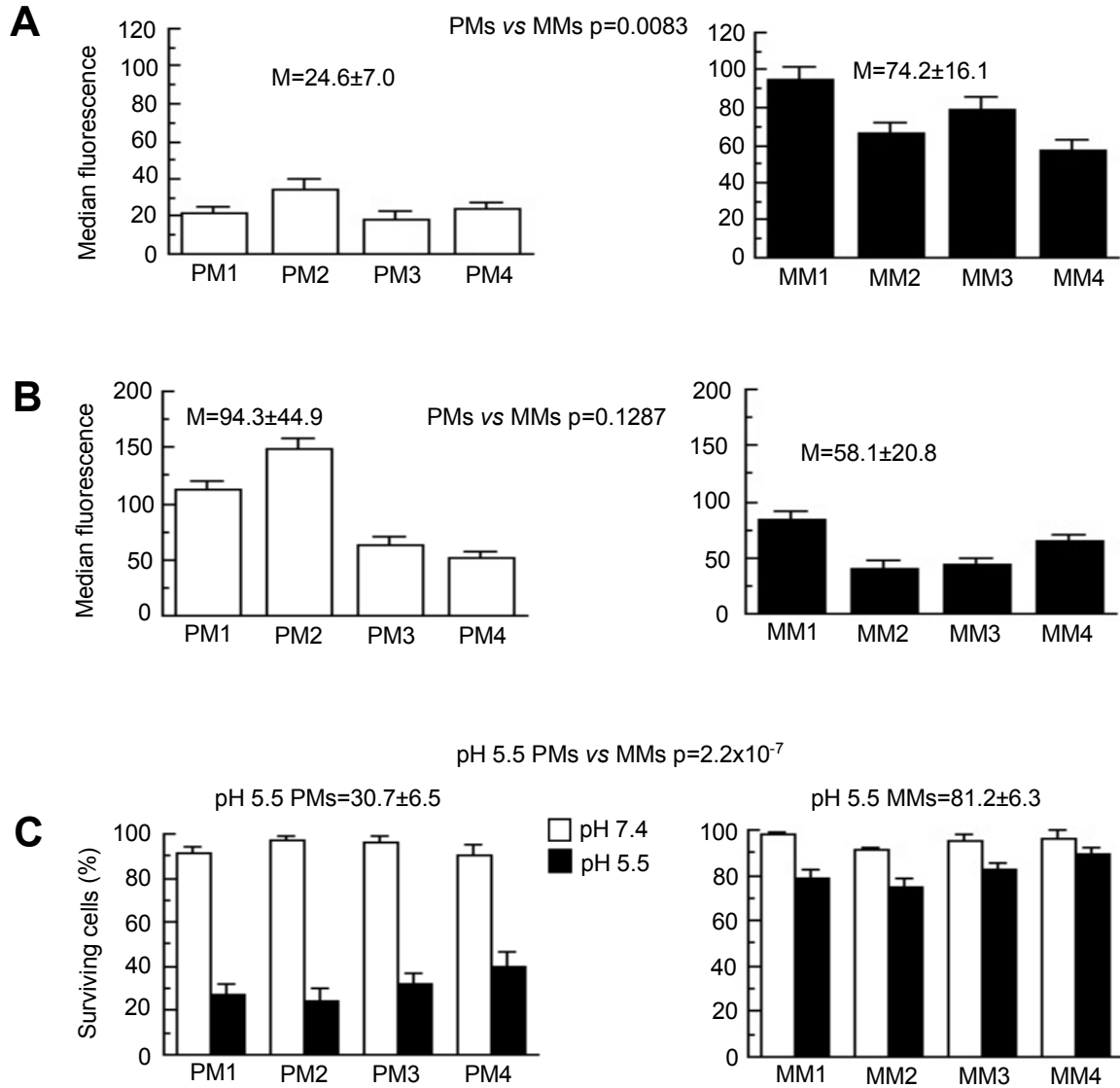

Supplement: Additional file 2 — Analysis of the lysosomal compartment and cell survival in acidic environment. Cytofluorimetric evaluation of lysosomal acidity (A) and lysosomal volume (B) in four different primary (white columns) and metastatic (black columns) human melanoma cell lines by using LysoSensor-green and LysoTracker-green dye, respectively. Data are reported as mean ± SD of the median fluorescence values among four independent experiments. Statistical analysis by Student's t-test indicates: p = 0.0083 for lysosomal acidity and p = 0.1287 for lysosomal volume for PM cell lines vs. MM cell lines. (C) Cell survival analysis at different pH values of the growth medium was performed by Trypan blue test. Data are reported as mean ± SD of the percentage of surviving cells obtained in three separate experiments performed in triplicate. Statistical analysis by Student's t-test indicates: p = 2.2 × 10^-7^ for PM cell lines vs. MM cell lines at pH 5.5. [file 1476-4598-9-207-S2.PDF]
